# Ten years of *Scientific Reports*

**DOI:** 10.1038/s41598-021-92477-5

**Published:** 2021-06-30

## Abstract

*Scientific Reports* launched in June 2011 with an inclusive ethos, and a mission to publish high-quality research without selecting papers based on perceived impact or significance. We reflect on our first 10 years, and thank our authors, reviewers and Editorial Board Members for their contributions to the success of the journal.

The vast majority of research involves small steps forward and it is not easy to predict which are steps towards major breakthroughs, or those that will later be found to have unexpected importance. Science is driven forward by these incremental advances in knowledge and understanding, and it progresses more rapidly when these contributions are disseminated quickly. But some research still goes completely unpublished, and overall progress is impeded when valid results are not made widely available. Researchers can find themselves sitting on results because they don’t fit the hypothesis, leading to publication bias; or due to the way researchers are assessed, they focus only on the results they think may be accepted by a selective journal.

We set out to serve the research community by providing a high-visibility, multidisciplinary Open Access journal that publishes scientifically robust original contributions, but where the community determines the value and importance of the work after publication. Since 2011, Open Access publishing has become more mainstream and there is increased understanding of the benefits it offers. There is also wider acknowledgement of the publishing option provided by a broad scoped, inclusive journal, and greater appreciation of how we can serve the broader research enterprise by improving research dissemination, reducing publication bias, and making the overall editorial and peer-review process more efficient and constructive. As we look back on our first 10 years, we are humbled by the number, breadth and diversity of the researchers who see the value in our approach and choose to publish with us.

To mark our 10th anniversary our in-house editors interviewed some our Editorial Board Members (EBMs), some of whom have been with us since the start, and others who have joined us more recently as Collection Guest Editors and Registered Reports EBMs. In these interviews, they reflect on their experiences at *Scientific Reports*, how the journal is achieving its mission, and its role in a changing research and publishing landscape.

Our EBMs allude to some positive signs that those assessing researchers are not relying on Journal Impact Factor to the same extent that they used to, and there are indications of a growing appreciation that negative results ought to be published, even if they’re still mostly not. But while there are reasons to be positive about this progress, we still face the fundamental problem that the research, publishing and academic systems do a poor job of rewarding rigour.

What comes across most strongly in the EBMs’ interviews is their commitment to the ethos of the journal, providing constructive peer review, and serving their communities. Of course the role is not without its challenges; there are the day-to-day issues of finding reviewers and managing peer review—something we have been focussing on by introducing new submission technology to improve author experience, and facilitate innovation in peer review. But there are also broader issues in that there is still insufficient credit, in terms of career development, for doing editorial work and reviewing papers.

We know that the COVID-19 pandemic continues to profoundly affect many of our contributors in terms of personal challenges, disruption and loss. It has also led to many researchers being busier than ever:  our EBM Sara Weibel explains how she has redirected her research to combat COVID-19. But there is hope that the pandemic may also help to reshape research in the long-term by encouraging early sharing of data, and release of early versions of work as pre-prints—things we have championed at *Scientific Reports* with our *Under Consideration* site for COVID-19 related work submitted to the journal.

We started out covering the natural sciences, expanding into clinical sciences, and more recently further expanding to cover engineering disciplines. The broad, multidisciplinary nature of the journal has helped to develop connections between different fields and foster collaborations to address global challenges and Sustainable Development Goals. Matjaž Perc, who works in social physics, explains how research is becoming more multidisciplinary, and that new fields now generally emerge at the interfaces of other fields. *Scientific Reports* has always been supportive and inclusive of such communities, helping them to grow and become more visible.

Florian Frommlet is Guest Editor of our ‘Improving reproducibility in animal research’ collection and he explains how multidisciplinary collaborations can also lead to increased rigour, and how he has seen a changing attitude to reproducibility issues over the last 10 years. To support reproducibility, we will introduce further initiatives and policies to improve reporting and transparency, but Florian explains why it is also important to identify and address the specific issues affecting different research subcultures. We recently introduced Registered Reports, which can promote rigour and combat publication bias, and we are encouraging their broader use.

We have also taken our 10th anniversary as an opportunity to select some of our favourite Collections of papers and authors’ stories. Our Collections allow us to support and highlight specific communities, to promote work that address Sustainable Development Goals, to react to events and respond to emerging fields of research. Our authors tell the stories of how their paper came into being, often through embracing multidisciplinary collaborations, benefiting from a constructive editorial and peer review process, and then finding that the published work had unexpected reach and impact.

We would like to finish by offering our profound thanks to our authors, reviewers and EBMs. We are hugely grateful for your contributions to the journal during its first 10 years, and we look forward to collaborating further for the good of the broad research community and research dissemination in general.

